# Paradox of Protection: Preferential Recognition of CD4-induced Epitopes by Anti-HIV-1 ADCC Antibodies

**DOI:** 10.1016/j.ebiom.2015.09.048

**Published:** 2015-09-30

**Authors:** Matthew S. Parsons, Stephen J. Kent

**Affiliations:** a Department of Microbiology and Immunology at the Peter Doherty Institute for Infection and Immunity, University of Melbourne, Melbourne, Australia; b Melbourne Sexual Health Centre, Alfred Health, Central Clinical School, Monash University, Melbourne, Australia; c ARC Centre of Excellence in Convergent Bio-Nano Science and Technology, University of Melbourne, Parkville, Australia

Antibodies capable of eliminating HIV-1-infected cells via antibody-dependent cellular cytotoxicity (ADCC) are present in most HIV-1-infected individuals. The ability of antibodies to trigger ADCC is associated with slower progression to AIDS, as well as vaccine-conferred protection from HIV-1 infection in the RV144 trial (Baum, L. L., et al., 1996, Haynes, B. F., et al., 2012). The RV144 trial enrolled over 16,000 subjects in Thailand. Half of the subjects were immunized with a prime-boost vaccine regimen that, although did not induce significant anti-HIV CD8 T cell or broad neutralizing antibody responses, did induce robust non-neutralizing antibody responses with ADCC function. Although ADCC antibodies can target a wide array of epitopes within the HIV-1 envelope (Env), recent studies have demonstrated that epitopes revealed within Env upon entering the CD4-bound conformation are preferentially targeted by ADCC antibodies within HIV-1-infected individuals and RV144 vaccinees (Bonsignori, M., et al., 2012, Veillette, M., et al., 2015). The HIV-1 Env protein binds to the cell-surface molecule CD4 to allow virion entry and this CD4 binding causes a conformational change in Env. In the current issue of *EBioMedicine*, Williams et al. provide further evidence of preferential targeting of the CD4-bound conformation of Env by ADCC antibodies in a Kenyan cohort infected with HIV-1 (Williams et al., 2015).

In their manuscript, Williams et al. demonstrate that GFP-tagged virus-like particles can be employed to identify and sort Env-specific B-cells (Williams et al., 2015). These B-cells' heavy and light chain immunoglobulin genes were sequenced then HIV-1-specific antibodies were generated and screened for anti-viral functions, such as ADCC. Utilizing this technology, Williams et al. identified three ADCC antibodies from an HIV-1 subtype A-infected donor. Two antibodies recognized epitopes revealed within the CD4-bound conformation of Env, while one recognized an epitope within the V3 loop of Env. Despite identifying an anti-V3 antibody capable of mediating ADCC, the anti-V3 specificity contributed very little to the ADCC response of the donor's plasma. Indeed, when the authors introduced mutations abrogating Fc receptor binding (i.e., LALA mutations) within each of the three monoclonal antibodies and used the mutants to block ADCC triggered by whole plasma, they observed robust inhibition by the LALA versions of the two antibodies specific for CD4-induced epitopes and no inhibition by the LALA version of the anti-V3 monoclonal antibody. To establish that CD4-induced epitopes were commonly targeted amongst the Kenyan cohort of the monoclonal antibody donor, the authors further demonstrated that LALA versions of the two antibodies specific for CD4-induced epitopes robustly inhibited the plasma ADCC of nine additional donors. These results are consistent with previous research demonstrating that ADCC-competent anti-variable loop antibodies are relatively rare amongst monoclonal antibodies isolated from RV144 vaccinees (Bonsignori et al., 2012). Further, this is consistent with an absorption experiment demonstrating that the majority of ADCC antibodies contributing to plasma ADCC responses are not directed to variable loop epitopes (Veillette et al.. 2015). Collectively, the data from Williams et al. and others demonstrates that, while some anti-V3 antibodies may be able to trigger ADCC, these antibodies contribute little to the capacity of plasma to clear infected cells via ADCC. Further research into the ability of anti-V3 antibodies to trigger ADCC, however, may prove fruitful for identifying ADCC antibodies capable of recognizing HIV-1 Env independently of CD4.

Much evidence points towards a role for Vpu and Nef in the evasion of ADCC responses by HIV-1-infected cells (Veillette et al.. 2014). Both Vpu and Nef downregulate cell surface CD4 and prevent Env from entering the CD4-bound conformation prominently targeted by ADCC antibodies (Veillette, M., et al., 2014, Veillette, M., et al., 2015). Vpu also downregulates cellular expression of tetherin, a protein involved in keeping HIV-1 virions at the cell surface and thus increasing epitope availability (Van Damme et al., 2008). Additionally, motifs within the membrane proximal region of Env serve to limit Env expression on the surface of infected cells (Von Bredow et al., 2015).

The Vpu and Nef-mediated down regulation of CD4 at first blush appears paradoxical to the notion that ADCC antibodies targeting the CD4-bound conformation of Env protect from HIV-1 infection. In their study, Williams et al. demonstrated that their two monoclonal antibodies specific for CD4-induced epitopes can inhibit HIV-1 replication in a CD4 + T cell line, CEM.NKr-CCR5 (Williams et al., 2015). These data demonstrate that these monoclonal antibodies can target the Env of the utilized isolate as it naturally appears on the surface of infected cells. Questions remain, however, about the competency of the Nef and Vpu of the viral isolate utilized, as well as the extent of CD4 downregulation on CEM.NKr-CCR5 cells infected with the viral isolate. An important research priority in this field is to identify epitopes and mechanisms whereby ADCC antibodies can target HIV-infected cells for elimination, regardless of viral Env conformation.

There is much hope that the mechanism of ADCC can, beyond the 31% efficacy of the RV144 trial, be exploited for prevention of HIV-1 infection through vaccination (Wren et al., 2013). Further, ADCC may assist in effort to develop a cure for HIV-1 infection through eliminating reactivated latently infected cells, since reactivating latently infected cells alone, without immune clearance, may be insufficient (Lee, W. S., et al., 2015, Wren, L. H., et al., 2013). To realize these goals there is likely a need to identify and utilize ADCC antibodies capable of recognizing non-CD4-bound Env epitopes, such as epitopes within V3, which would allow for the elimination of latent viruses or transmitted/founder viruses that fully or sufficiently downregulate CD4 and render ADCC antibodies targeting CD4-induced epitopes non-functional. A major caveat of targeting the V3 region is the high diversity in this region between viruses. Importantly, Williams et al. provide proof of principle that epitopes present on Env not bound to CD4 can be targeted for ADCC by antibodies specific for CD4-independent epitopes (Williams et al., 2015).

## Conflicts of interest

The authors declare no conflicts of interest.
